# The Genomic Signature of Breast Cancer Prevention

**DOI:** 10.3390/genes5010065

**Published:** 2014-02-26

**Authors:** Jose Russo, Julia Santucci-Pereira, Irma H. Russo

**Affiliations:** The Irma H. Russo MD Breast Cancer Research Laboratory, Fox Chase Cancer Center, Temple University Health System, 333 Cottman Avenue, Philadelphia, PA 19111, USA; E-Mail: Julia.Pereira@fccc.edu

**Keywords:** normal breast, breast cancer, genomic signature, prevention, pregnancy, splicing mechanisms, methylation, chromatin remodeling, Lnc-RNA, beta-catenin

## Abstract

The breast of parous postmenopausal women exhibits a specific signature that has been induced by a full term pregnancy. This signature is centered in chromatin remodeling and the epigenetic changes induced by methylation of specific genes which are important regulatory pathways induced by pregnancy. Through the analysis of the genes found to be differentially methylated between women of varying parity, multiple positions at which beta-catenin production and use is inhibited were recognized. The biological importance of the pathways identified in this specific population cannot be sufficiently emphasized because they could represent a safeguard mechanism mediating the protection of the breast conferred by full term pregnancy.

## 1. Introduction

More than 300 years ago, an excess in breast cancer mortality in nuns was reported, in whom the increased risk was attributed to their childlessness [1] until MacMahon *et al*. [2] found an almost linear relationship between a woman’s risk and the age at which she bore her first child. This work confirmed that pregnancy had a protective effect that was evident from the early teen years and persisted until the middle twenties [1]. Other studies have reported that additional pregnancies and breastfeeding confer greater protection to young women, including a statistically significantly reduced risk of breast cancer in women with deleterious BRCA1 mutations who breast-fed for a cumulative total of more than one year [3,4]. Our studies, designed to unravel what specific changes occurred in the breast during pregnancy that confer a lifetime protection from developing cancer, led us to the discovery that endogenous endocrinological or environmental influences affecting breast development before the first full term pregnancy were important modulators of the susceptibility of the breast to undergo neoplastic transformation. The fact that exposure of the breast of young nulliparous females to environmental physical agents [5] or chemical toxicants [6,7] results in a greater rate of cell transformation suggests that the immature breast possesses a greater number of susceptible cells that can become the site of the origin of cancer, similarly to what has been reported in experimental animal models [8,9,10,11]. In these models, the initiation of cancer is prevented by the differentiation of the mammary gland induced by pregnancy [11,12]. The molecular changes involved in this phenomenon are just starting to be unraveled [13,14,15,16,17,18]. The protection conferred by pregnancy is age-specific since a delay in childbearing after age 24 progressively increases the risk of cancer development. Eventually, this risk becomes greater than that of nulliparous women when the first full term pregnancy (FFTP) occurs after 35 years of age [2]. The higher breast cancer risk which has been associated with early menarche further emphasizes the importance of the length of the susceptibility “window” that encompasses the period of breast development occurring between menarche and the first pregnancy, when the organ is more susceptible to undergo complete differentiation under physiological hormonal stimuli. Differentiation is a hallmark that protects the breast from developing cancer by lessening the risk of suffering genetic or epigenetic damages. This postulate is supported by our observations that the architectural pattern of lobular development in parous women with cancer differs from that of parous women without cancer; the former being similar to the architectural pattern of lobular development of nulliparous women with or without cancer. Thus, the higher breast cancer risk in parous women might have resulted from either a failure of the breast to fully differentiate under the influence of the hormones of pregnancy and/or proliferation of transformed cells initiated by early damage or genetic predisposition [18].

Numerous studies have been performed to understand how the dramatic modifications that occur during pregnancy in the pattern of lobular development and differentiation, cell proliferation, and steroid hormone receptor content of the breast influence cancer risk [18]. Studies at the molecular level using different platforms for global genome analysis have confirmed the universality of this phenomenon in various strains of rats and mice [13,14,15,16,17,18,19,20,21]. Studies in experimental animal models have been useful for uncovering the sequential genomic changes occurring in the mammary gland in response to multiple hormonal stimuli of pregnancy that lead to the imprinting of a permanent genomic signature. Our results support our hypothesis that post-menopausal parous women exhibit a genomic “signature” that differs from the expression present in the breast of nulliparous women, who traditionally represent a high breast cancer risk group.

## 2. Phenotypic Changes Induced by Pregnancy in the Human Breast

Our study has been done using core biopsies of nulliparous (NP) and parous (P) postmenopausal women [22,23]. The nulliparous group included both nulligravida nulliparous (NN) and gravida nulliparous (GN); both NN and GN women were considered within the NP as a single group for most analyses, unless indicated otherwise. Our previous studies have in great part clarified the role of pregnancy-induced breast differentiation in the reduction in breast cancer risk, as well as the identification of lobules type 1 (Lob 1) or the terminal ductal lobular unit (TDLU) as the site of origin of breast cancer [4,7,24]. The morphological, physiological and genomic changes resulting from pregnancy and hormonally-induced differentiation of the breast and their influence on breast cancer risk have been addressed in previous publications [4,7,24,25]. Our observations that during the post-menopausal years the breast of both parous and nulliparous women contains preponderantly Lob 1, and the fact that nulliparous women are at higher risk of developing breast cancer than parous women, indicate that Lob 1 in these two groups of women either differ biologically, or exhibit different susceptibility to carcinogenesis [25]. The breast tissues of the P and NP women contained ducts and Lob 1 [4,12,26]. 

The microscopic analysis of the breast tissue revealed that the population of luminal cells lining ducts and Lob 1 was composed of cells that were characterized by their nuclear appearance into two types: one that contained large and palely stained nuclei with prominent nucleoli and another consisting of small hyper chromatic nuclei [27]. The pale staining of the large former nuclei is a feature indicative of a high content of non-condensed euchromatin; these nuclei were called euchromatin-rich nuclei (EUN). The hyperchromasia observed in the latter nuclei was indicative of chromatin condensation and high content of heterochromatin; these nuclei were identified as heterochromatin-rich nucleus (HTN). The analysis of the distribution of HTN and EUN cells in histological sections of the breast core biopsies revealed that EUN were more abundant in the NP than in the P breast tissues, whereas the inverse was true for the HTN; these differences were statistically significant [27]. We have confirmed the differences between the HTN and EUN using a quantitative image analysis system [27]. The nuclear size (diameter, area and perimeter) of the EUN as a whole was significantly higher (*p <* 0.05) than that of the HTN in both nulliparous and parous women. Differences were also found to be statistically significant (*p <* 0.05) regarding the nuclear shape (nuclear feret ratio) in the breast of nulliparous women, indicating that in these breasts the nuclei of the HTN had a more elongated ellipsoidal shape than the EUN. The light absorbance (mean gray values/nucleus) was always greater for EUN than for HTN of both NP and P breasts, either considered as two groups or individually, an indication that under densitometric terms HTN were always more densely stained than EUN. Comparison of the EUN of nulliparous *vs*. parous breasts revealed significant differences in nuclear size, stainability and densitometric energy, leading us to conclude that epithelial cell nuclei were larger, less stainable and with smaller regions with uniform densitometric intensity in nulliparous breasts. Comparison of the HTN of nulliparous *vs*. parous breasts revealed significant differences in nuclear diameter, perimeter, shape and stainability; cell nuclei showed larger contours and more elongated ellipsoidal shape and they were more stainable in nulliparous breasts. These observations indicated that a shift of the EUN cell population to a more densely packed chromatin cell (HTN) had occurred in association with the history of pregnancy as a distinctive pattern of the postmenopausal parous breast [27].

Since chromatin condensation is part of the process of chromatin remodeling towards gene silencing that is highly regulated by methylation of histones, we verified this phenomenon by immunohistochemistry (IHC) incubating NP and P breast tissues with antibodies against histone 3 dimethylated at lysine 9 (H3K9me2) and trimethylated at lysine 27 (H3K27me3) [27]. The IHC stain revealed that methylation of H3 at both lysine 9 and 27 was increased in the heterochromatin condensed nuclei of epithelial cells of the parous breast when compared to the euchromatin rich nuclei of the nulliparous breast. In the nulliparous breast, the reactivity in individual cells was less intense and the number of positive cells was significantly lower. These variations in chromatin reorganization were supported by the upregulation of CBX3, CHD2, L3MBTL, and EZH2 genes controlling this process (Table 1) [27].

**Table 1:** Genes upregulated in the parous breast.

Symbol	Log Ratio	*P* value	Gene Name
Apoptosis (GO:0006915; GO:0006917; GO:0008624; GO:0042981)			
CASP4	0.37	0.0003	caspase 4, apoptosis-related cysteine peptidase
RUNX3	0.36	0.0000	runt-related transcription factor 3
LUC7L3	0.34	0.0002	LUC7-like 3 (S. cerevisiae)
ELMO3	0.30	0.0003	engulfment and cell motility 3
DNA repair (GO:0006281; GO:0006284)			
SFPQ	0.46	0.0002	splicing factor proline/glutamine-rich
MBD4	0.36	0.0003	methyl-CpG binding domain protein 4
RBBP8	0.32	0.0000	retinoblastoma binding protein 8
Cell adhesion (GO:0007155; GO:0030155)			
NRXN1	0.60	0.0001	neurexin 1
DSC3	0.51	0.0000	desmocollin 3
COL27A1	0.44	0.0002	collagen, type XXVII, alpha 1
PNN	0.37	0.0001	pinin, desmosome associated protein
COL4A6	0.36	0.0008	collagen, type IV, alpha 6
LAMC2	0.34	0.0008	laminin, gamma 2
COL7A1	0.33	0.0002	collagen, type VII, alpha 1
COL16A1	0.31	0.0000	collagen, type XVI, alpha 1
LAMA3	0.30	0.0008	laminin, alpha 3
Cell cycle (GO:0000075; GO:0007049; GO:0045786)			
SYCP2	0.45	0.0000	synaptonemal complex protein 2
PNN	0.37	0.0001	pinin, desmosome associated protein
RUNX3	0.36	0.0000	runt-related transcription factor 3
RBBP8	0.32	0.0000	retinoblastoma binding protein 8
Cell differentiation (GO:0001709; GO:0030154; GO:0030216)			
MGP	0.53	0.0003	matrix Gla protein
KRT5	0.41	0.0002	keratin 5
GATA3	0.35	0.0009	GATA binding protein 3
LAMA3	0.30	0.0008	laminin, alpha 3
Cell proliferation (GO:0008283; GO:0008284; GO:0008285; GO:0042127; GO:0050679; GO:0050680)			
PTN	0.67	0.0002	Pleiotrophin
KRT5	0.41	0.0002	keratin 5
RUNX3	0.36	0.0000	runt-related transcription factor 3
IL28RA	0.34	0.0003	interleukin 28 receptor, alpha (interferon, lambda receptor)
CDCA7	0.31	0.0005	cell division cycle associated 7
Cell motility (GO:0006928; GO:0030334)			
DNALI1	0.37	0.0001	dynein, axonemal, light intermediate chain 1
LAMA3	0.30	0.0008	laminin, alpha 3
G-protein coupled receptor pathway (GO:0007186)			
OXTR	0.54	0.0006	oxytocin receptor
RNA metabolic process (GO:0000398; GO:0001510; GO:0006376; GO:0006396; GO:0006397; GO:0006401; GO:0008380)			
METTL3	0.69	0.0000	methyltransferase like 3
HNRPDL	0.65	0.0001	heterogeneous nuclear ribonucleoprotein D-like
HNRNPD	0.59	0.0003	heterogeneous nuclear ribonucleoprotein D (AU-rich element RNA binding protein 1, 37 kDa)
HNRNPA2B1	0.56	0.0003	heterogeneous nuclear ribonucleoprotein A2/B1
SFPQ	0.47	0.0006	splicing factor proline/glutamine-rich
RBM25	0.38	0.0009	RNA binding motif protein 25
RBMX	0.38	0.0000	RNA binding motif protein, X-linked
LUC7L3	0.34	0.0002	LUC7-like 3 (S. cerevisiae)
SFRS1	0.30	0.0001	splicing factor, arginine/serine-rich 1
RNA transport (GO:0050658)			
HNRNPA2B1	0.56	0.0003	heterogeneous nuclear ribonucleoprotein A2/B1
Transcription (GO:0006350; GO:0006355; GO:0006357; GO:0006366; GO:0016481; GO:0045449; GO:0045893; GO:0045941)			
HNRPDL	0.65	0.0001	heterogeneous nuclear ribonucleoprotein D-like
HNRNPD	0.59	0.0003	heterogeneous nuclear ribonucleoprotein D (AU-rich element RNA binding protein 1, 37 kDa)
CBX3	0.53	0.0003	chromobox homolog 3 (HP1 gamma homolog, Drosophila)
NFKBIZ	0.48	0.0001	nuclear factor of kappa light polypeptide gene enhancer in B-cells inhibitor, zeta
FUBP1	0.47	0.0002	far upstream element (FUSE) binding protein 1
SFPQ	0.47	0.0006	splicing factor proline/glutamine-rich
EZH2	0.44	0.0000	enhancer of zeste homolog 2 (Drosophila)
ZNF207	0.41	0.0007	zinc finger protein 207
ZNF711	0.41	0.0003	zinc finger protein 711
GATA3	0.38	0.0009	GATA binding protein 3
PNN	0.37	0.0003	pinin, desmosome associated protein
ZNF107	0.37	0.0001	zinc finger protein 107
RUNX3	0.36	0.0000	runt-related transcription factor 3
CCNL1	0.35	0.0009	cyclin L1
ZNF692	0.34	0.0000	zinc finger protein 692
CHD2	0.33	0.0001	chromodomain helicase DNA binding protein 2
RBBP8	0.32	0.0000	retinoblastoma binding protein 8
ZNF789	0.32	0.0005	zinc finger protein 789
CDCA7	0.31	0.0005	cell division cycle associated 7
Chromatin organization (GO:0006333; GO:0006338)			
CBX3	0.53	0.0003	chromobox homolog 3 (HP1 gamma homolog, Drosophila)
CHD2	0.33	0.0001	chromodomain helicase DNA binding protein 2
Cell division (GO:0051301)			
SYCP2	0.45	0.0000	synaptonemal complex protein 2
DNA metabolic process (GO:0006139; GO:0006260; GO:0006310; GO:0015074)			
METTL3	0.69	0.0000	methyltransferase like 3
SFPQ	0.46	0.0002	splicing factor proline/glutamine-rich
GOLGA2B	0.32	0.0001	golgin A2 family, member B
Lactation (GO:0007595)			
OXTR	0.54	0.0006	oxytocin receptor

## 3. Transcriptomic Differences Induced by Pregnancy

Analysis of P and NP gene expression microarrays revealed that there were 305 probe sets, corresponding to 208 distinct genes, differentially expressed between these two groups. Of the 305 probe sets, 267 were up- and 38 were down-regulated [22,23]. From these 267 up-regulated genes, we described biological processes that were representative of the transcriptomic differences between the parous and the nulliparous breasts. Using bioinformatics based analysis of microarray data, we found that the biological processes involving the splicing machinery and mRNA processing were prevalent in the parous breast and were represented by the following upregulated genes: LUC7L3, SFRS1, HNRNPA2B1, HNRNPD, RBM25, SFRS5, METTL3, HNRNPDL, and SFPQ (Table 1). Transcription regulation and chromatin organization were also highly represented in the parous breast by the upregulation of CBX3, EBF1, GATA3, RBBP8, CCNL1, CCNL2, CDCA7, EZH2, FUBP1, NFKBIZ, RUNX3, ZNF107, ZNF207, ZNF692, ZNF711, ZNF789, CDCA7, and ZNF692 (Table 1). The parous breast also expressed upregulation of six non-coding regions that included XIST, MALAT-1 (or NEAT2) and NEAT1 [27].

Genes that were down-regulated in the parous breast represented transcription regulation, encompassing CBL, FHL5, NFATC3, NCR3C1, TCF7L2, and a set of genes that were involved in IGF-like growth factor signaling, somatic stem cell maintenance, muscle cell differentiation and apoptosis, such as IGF1, RASD1, EBF1, SOX 1,SOX6, SOX 17, RALGAPA2 and ABHD5. In rodents, also was observed the reduction of expression of genes related to growth factors, such as Igf1 [15]. The level of expression was confirmed to be differentially expressed between nulliparous and parous breast tissues by real time RT-PCR for the following genes: CREBZF, XIST, MALAT1, NEAT1, CCNL2, GATA3, DDX17, HNRPDL, SOX6, SNHG12, SOX 17 and C1orf168 [23]. In addition to the level of expression, the localization of the alternative splicing regulator cyclin L2 protein (CCNL2) [28], was verified by IHC. CCNL2 protein was expressed in the nucleus of epithelial cells in breast tissues from NP and P women, although the level of expression was significantly higher in Lob 1 in the parous breast when compared with similar structures found in the breast of nulliparous women. These observations confirmed the localization of this gene product in the splicing factor compartment (nuclear speckles) [29].

## 4. Shifting of the Cell Population in the Human Breast

We found a shift in the cell population of the postmenopausal breast as a manifestation of the reprogramming of the organ after pregnancy. These observations are in agreement with what is observed in the rat mammary gland, which also contains two types of luminal epithelial cells, designated dark (DC) and intermediate (IC) cells, in addition to the myoepithelial cells [30]. The DC and IC are equivalent to the HTN and EUN cells described in the present work. DCs increase after pregnancy and lactational involution; whereas the ICs significantly outnumber the DC in ductal hyperplasias and ductal carcinomas [30,31]. Our analysis of nuclear ultrastructural and morphometric parameters of rodent IC have allowed us to differentiate the mammary progenitor stem cell from the cancer stem cells [25,30,31]. Nuclear morphometric analysis of breast and ovarian carcinomas has confirmed the predictive value of nuclear grade on the progression of premalignant lesions to invasiveness [32,33,34]. Our findings of a significant decrease in the number of EUN with a subsequent increase in the number of HTN cells expressing specific biomarkers identified at the chromatin and transcriptional levels support the value of morphometric analysis as an adjuvant to molecular studies [27]. Our data clearly indicate that there are morphological indications of chromatin remodeling in the parous breast, such as the increase in the number of epithelial cells with condensed chromatin and increased reactivity with anti-H3K9me2 and H3K27me3 antibodies. Histone methylation is a major determinant for the formation of active and inactive regions of the genome and is crucial for the proper programming of the genome during development [35]. In the parous breast, there is upregulation of transcription factors and chromatin remodeling genes such as CHD2 or chromodomain helicase DNA binding protein 2 and the CBX3 or Chromobox homolog 3, whose products are required for controlling recruitment of protein/protein or DNA/protein interactions. CBX3 is involved in transcriptional silencing in heterochromatin-like complexes, and recognizes and binds H3 tails methylated at lysine 9, leading to epigenetic repression. Two other important genes related to the polycomb group (PcG) protein that are upregulated in the parous breast are the L3MBTL gene or l(3)mbt-like and the histone-lysine N-methyltransferase or EZH2. Members of the PcG form multimeric protein complexes that maintain the transcriptional repressive state of genes over successive cell generations (Table 1). EZH2 is an enzyme that acts mainly as a gene silencer, performing this role by the addition of three methyl groups to lysine 27 of histone 3, a modification that leads to chromatin condensation [30,36,37].

## 5. Methylation Changes in the DNA of Parous Women are Part of Chromatin Remodeling and the Genomic Signature of Pregnancy

**Figure 1:**
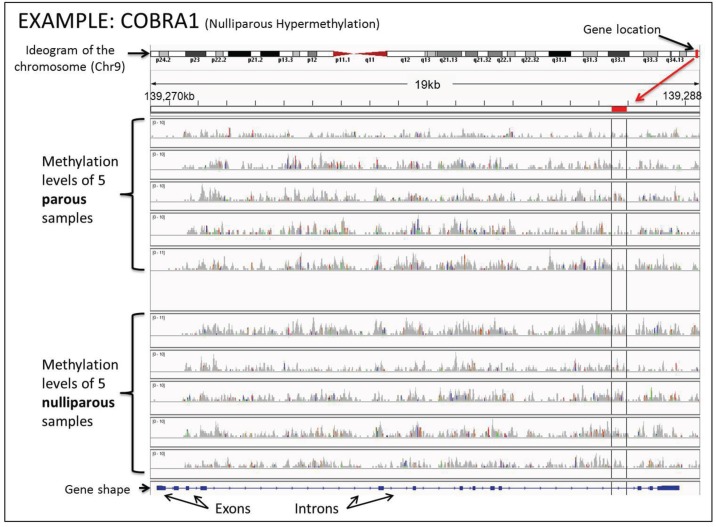
Overview of how the DNA methylation levels appear in the Integrative Genomics Viewer (IGV). At the top of the figure is the ideogram of the chromosome given by IGV, with the area currently being examined marked in red. At the bottom is the overall shape of the gene containing exons and introns. Exons are shown as thicker blue sections on the overall gene. The gray bars represent the methylation levels of each volunteer at each base pair. They are created by combining each read resulting from the sequencing done on the samples. The higher they are, the higher the percentage of methylation is at any given base pair. When there was an area of higher methylation occurring in at least four of the members of one parity group as compared to all members of the opposing group, that area was defined as a differentially methylated region (DMR).

**Figure 2:**
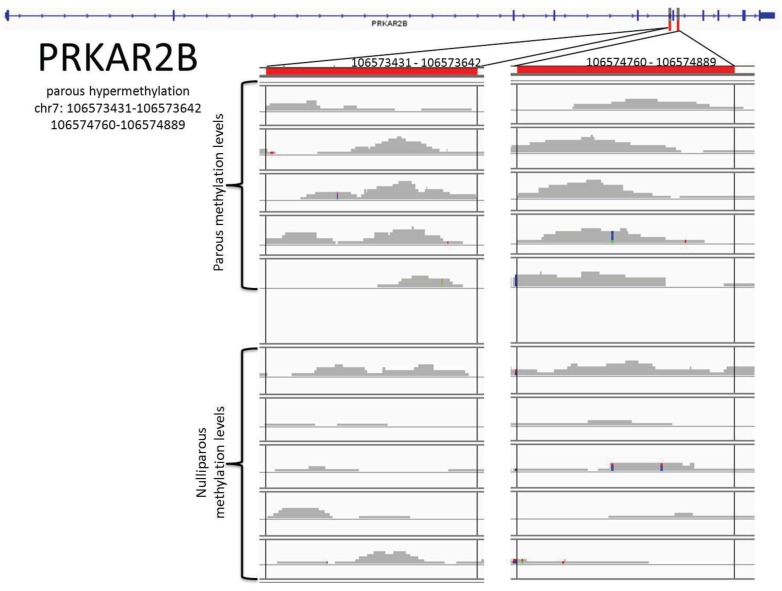
DMRs for PRKAR2B. At the top we see the gene shape, with the red marked DMRs. Any colored locations within the gray bars indicate a nucleotide read which is different from the reference genome.

The chromatin remodeling process is demonstrated not only by the shifting of the EUN to the HTN cells, but also confirmed by the increase in methylation of histones H3K9me2 and H3K27me3. This is an indication that methylation of other genes could also be involved in the process. Using the DNA from five nulliparous and five parous breast core biopsies and applying the MBD-cap sequencing methodology [38], we have identified 583 genes showing different levels of methylation between the parous and nulliparous breasts. From the 583 genes, 455 were hypermethylated in the parous while 128 were hypermethylated in the nulliparous breast, confirming the reprogramming of the chromatin to a more silenced or resting stage. To get a better understanding of the methylation profile of the 583 genes, we used Integrative Genomics Viewer (IGV) software [39,40]. IGV was utilized to identify the distinct areas, throughout the entire gene, where the methylation levels differed between the sample groups. The identification of these areas, known as differentially methylated regions (DMRs), is important because they are more likely to affect gene expression [41]. We performed the comparison between the nulliparous and parous methylation profiles against the human reference genome “hg 18” and against each other. For example, the gene COBRA 1, which is the cofactor of BRCA1 and has been shown to work in its regulatory pathway [42], was hypermethylated in the nulliparous breast. It is shown in Figure 1 that the methylation levels for each sample at each base pair that an area of higher methylation occurring in at least four of the samples of one group as compared to all members of the opposing group, that area was defined as a (DMR) (Figure 1 and Figure 2). COBRA1 had a DMR near the end of the gene, which was marked in Figure 1 using the IGV’s marking tool. When a differentially methylated area is found and marked, hovering over the red marker at the top of the sample area gives the exact chromosomal location. Every gene within the 583 gene list was closely examined for DMRs. The chromosomal locations at which these DMRs were found and marked were recorded in Table 2 and Table 3.

After analysis of the 583 genes using the IGV, we have identified the DMRs of 53 genes. Of the 455 parous hypermethylated genes, 41 had DMRs. These were NEGR1, NUF2, SYT14, POU4F1, FLRT2, ASAP2, DNAJC13, IFITM4P, ZNF292, SDK1, ELAVL4, DACT1, SPATA5L1, DYNC1I2, NLGN1, MAN1A1, AK5, DPYD, PROX1, PDE3A, NOVA1, SKAP1, ANKRD12, B4GALT5, CNTN4, ROBO1, GSK3B, INPP4B, FNIP2, IL6ST, TICAM2, PPP2CA, C6orf138, PRKAR2B, TTLL7, MAN1A2, CDC42BPA, OSBP, STIM2, NR3C2, and REV3L. The exact locations of these DMRs are recorded in Table 2. A point of interest within these genes is that DNAJC13 and GSK3B, while statistically given to be hypermethylated within parous women, had DMRs which suggested nulliparous hypermethylation. Because of this and for the scope of this experiment, those genes are treated as nulliparous hypermethylated. Of the 128 nulliparous hypermethylated genes, 12 had DMRs. These were NHSL2, PTX4, LRRC37A3, C20orf166-AS1, TPPP, NELF, SAMD10, CELSR1, FZD1, TNFRSF18, SRMS, and COBRA1. The chromosomal locations of these DMRs can be seen in Table 3. Within this list only C20orf166-AS1 was found to have a DMR in the direction opposite to what the statistics showed. Visual examples of these differentially methylated areas are seen in Figure 2 and Supplementary Figures S1–S4.

**Table 2:** DMRs within parous hypermethylated genes.

Parous Hypermethylated Genes		
NEGR1	chr1	71702567-71703327
		72142369-72142934
NUF2	chr1	161576182-161576653
SYT14	chr1	208309959-208310406
		208206495-208206910
POU4F1	chr13	78072725-78073146
FLRT2	chr14	85155301-85155789
ASAP2	chr2	9266977-9267464
		9432659-9433115
DNAJC13	Chr3	133712540-133712930
IFITM4P	Chr6	29826792-29827266
ZNF292	Chr6	88022117-88022631
SDK1	Chr7	4121961-4122279
		4230104-4230384
ELAVL4	Chr1	50387715-50388146
DACT1	Chr14	58182547-58182717
SPATA5L1	Chr15	43494615-43495210
DYNC1I2	Chr2	172279940-172280462
NLGN1	Chr3	175147546-175148159
		175156296-175156626
		175277928-175278476
MAN1A1	Chr6	119623891-119624320
AK5	Chr1	77616541-77616886
		77655265-77655548
DPYD	Chr1	98153997-98154252
PROX1	Chr1	212267523-212267905
PDE3A	Chr12	20432463-20432808
NOVA1	Chr14	26015695-26016215
SKAP1	Chr17	43591761-43592022
ANKRD12	Chr18	9168269-9168654
B4GALT5	Chr20	47704095-47704520
CNTN4	Chr3	2572819-2573349
ROBO1	Chr3	79026030-79023709
GSK3B	Chr3	121258375-121258501
INPP4B	Chr4	143292977-143293319
		143347212-143347585
		143966478-143966985
FNIP2	Chr4	159911129-159911596
		160015288-160015809
IL6ST	Chr5	55271135-55271466
TICAM2	Chr5	114955685-114955992
		114956473-114956938
PPP2CA	Chr5	133567556-133567871
C6orf138	Chr6	48025616-48025836
		48067151-48067418
PRKAR2B	Chr7	106573431-106573642
		106574760-106574889
TTLL7	Chr1	84185339-84185660
MAN1A2	Chr1	117816180-117816444
CDC42BPA	Chr1	225520202-225520399
OSBP	Chr11	59121100-59121437
		59121927-59122155
STIM2	Chr4	26572404-26572775
NR3C2	Chr4	149367631-149368052
REV3L	Chr6	111804054-111804285

**Table 3:** DMRs within nulliparous hypermethylated genes.

Nulliparous Hypermethylated Genes		
NHSL2	chrX	71270541-71271527
C16orf38 (PTX4)	Chr16	1476600-1476773
LRRC37A3	Chr17	60311872-60311982
C20orf200 (C20orf166-AS1)	Chr20	60557111-60557421
TPPP	Chr5	742334-742618
NELF	Chr9	139471353-139471653 (HYPO)
		139471653-139471895
SAMD10	Chr20	62077471-62077661
CELSR1	Chr22	45272965-45273071
FZD1	Chr7	90733372-90733621
TNFRSF18	Chr1	1130349-1130634
SRMS	Chr20	61646714-61647041
COBRA1	Chr9	139285424-139285977

**Figure 3:**
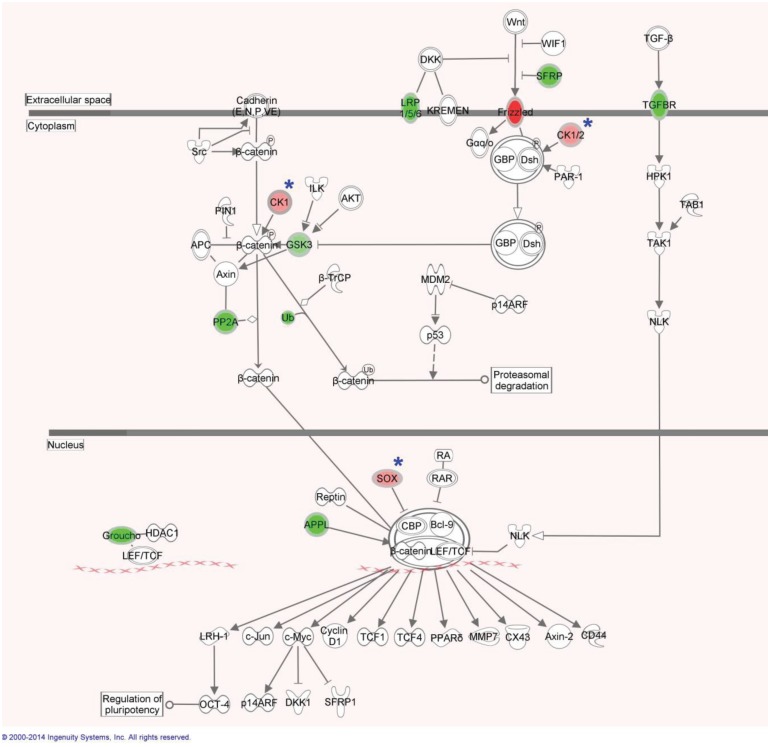
Canonical WNT/β-catenin signaling genes marked in green are hypermethylated in parous women (suggesting down-regulation of the gene in parous women). Genes in red are hypermethylated within nulliparous women. Genes marked with (*) were observed differentially expressed the microarray data. This canonical pathway was generated through the use of IPA (Ingenuity^®^ Systems) [43].

**Figure 4:**
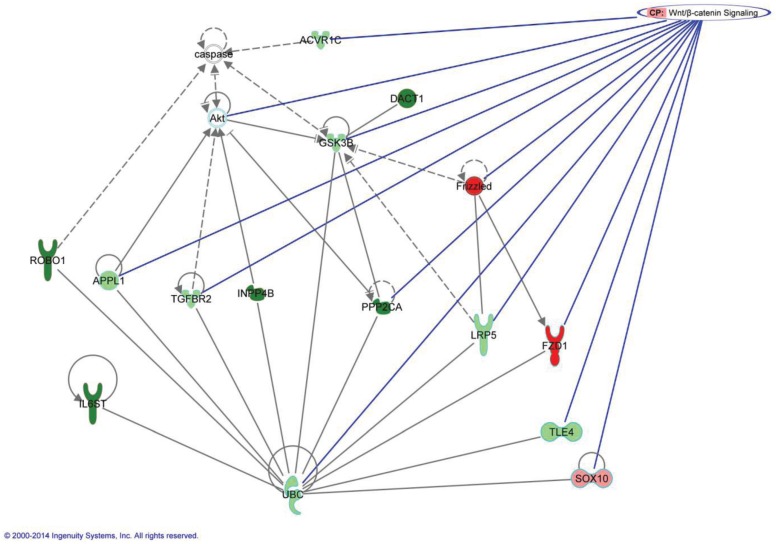
Interaction of target genes in Wnt/β-catenin signaling. The green genes are statistically parous hypermethylated, while the ones colored red are statistically nulliparous hypermethylated. The darker genes have recorded DMRs, and this is to the exception of GSK3B, which was first found statistically significant hypermethylated in the parous breast, but its DMR is hypermethylated in the nulliparous samples. This network was generated through the use of IPA (Ingenuity^®^ Systems) [43].

Analysis and research into the functions of these 53 genes identified seven which interacted with each other in either the Wnt signaling pathway or its controlling PI3K/AKT/mTOR pathways. The DMRs of these genes (DACT1, PPP2CA, GSK3B, ROBO1, INPP4B, IL6ST, FZD1) are shown in Supplementary Figures S1–S4. An overview of the involvement in the canonical Wnt pathway is shown in Figure 3. The interworking of these genes with each other and with other genes within the statistically methylated 583 can be seen in Figure 4.

Of the seven genes with DMRs which we have shown to work together in the Wnt pathway or its controllers, three worked directly in canonical Wnt signaling. Interestingly, when we analyzed the genes differentially expressed between parous and nulliparous [23], we found genes that also participate in the Wnt pathway, such as CSNK1A1 and SOX family (Figure 3). FZD1, which is the hypermethylated in the nulliparous breast, codes for the Frizzled receptor. When activated, this receptor directly activates Disheveled (Dsh) in the cytosol to begin the Wnt signaling cascade [44]. GSK3B, which also contains DMRs hypermethylated in the nulliparous women, has as main rule to decrease beta-catenin levels in the Wnt signaling pathway [45]. PPP2CA (PP2A) is suggested to work both upstream and downstream of beta-catenin to assist in its stabilization [46]. DACT1 assists in Wnt signaling by up-regulating GSK3B [47]. ROBO1, INPP4B and IL6ST genes are active in PI3K dependent AKT signaling [48,49,50].

The potential significance of the Wnt signaling pathway is rooted in an experiment performed in 1982 to find which genes would mutate in mice injected with mouse mammary tumor virus locating int1, a proto-oncogene [51]. Int1 was soon found to be highly conserved across multiple species, including drosophila and humans. Int1 was discovered to be the mammalian homologue of the drosophila Wingless (Wg), a gene previously found to be a segment polarity gene in embryonic development. The Wnt signaling pathway was given its name from the combination of Wg and int1, and has always had a close relationship to both differentiation and breast cancer.

Mammary development requires complex, reciprocal epithelial mesenchymal interactions. During embryonic development, Wnt signaling is involved in the initiation and early formation of mammary buds [52]. Then, during pregnancy, the pathway is activated to help the differentiation of mammary ducts in preparation for lactation. It does this by increasing beta-catenin levels in the cytosol and the nucleus, which in turn increases epithelial-mesenchymal transition and aids in transcription. After weaning, the mammary glands go through involution and the E-cadherin binding domain for beta-catenin is truncated [53]. This decreases cellular adhesion and signal epithelial apoptosis. The result is a lessened need for beta-catenin. In fact, overexpression of beta-catenin during involution results in a lack of complete involution [54]. This suggests that lowered beta-catenin expression is essential for proper mammary involution. Studies in mouse model systems clearly demonstrate that activated Wnt signaling leads to mammary tumorigenesis [55]. Misra *et al.* observed alteration in Fzd4 and Wnt2 expression in rats after full term pregnancy [20]. Other studies have shown an increase in cytosolic/nuclear beta-catenin in about 60% of breast cancers. This is usually explained by the pathway’s ability to aid in epithelial-mesenchymal transition and cell proliferation, two things incredibly important in the progression of cancer. Recently, the Wnt signaling pathway has been directly implicated in the parity induced protective effect against breast cancer [56]. It was revealed that parity induces differentiation and down-regulates the Wnt/Notch signaling ratio of basal stem/progenitor cells in mice. The down-regulation was attributed to a reduced expression of Wnt4, a necessary ligand in the activation stages of the Wnt pathway, in the mammary cells of parous mice [56]. 

The nulliparous hypermethylation of FZD1 suggests an up-regulation of the Frizzled family receptors and through this an up-regulation of all three types of Wnt signaling, indeed, we observed a slight overexpression of this gene in the parous women (not statistically significant). Increased Wnt signaling is associated with an increase in EMT in both development and cancer [57,58]. However, despite the Wnt signaling pathways being seemingly up-regulated, key genes within the pathways appear within our data to be down-regulated, thus changing the outcome of the signals sent through the Frizzled receptors. Signals sent through the Fz receptors activate the phosphoprotein Disheveled (Dsh). Dsh has three highly conserved protein domains, which interact differently depending on which Wnt pathway it is interacting with [44]. An up-regulation of FZD1 assumes an overall up-regulation of Dsh activation, and thus an increase in all three Wnt pathways. The three pathways are the canonical Wnt/beta-catenin pathway, the noncanonical planar cell polarity (PCP) pathway, and the noncanonical Wnt/calcium pathway.

The canonical pathway is the only one to involve beta-catenin, which is the TCF/LEF binding protein responsible for increased transcription and EMT [57,58]. Intracellular beta-catenin levels are maintained through constant creation and destruction, the processes of which are suggested to be regulated differently between our parity groups.

The canonical Wnt pathway contains the beta-catenin destruction complex, which is usually down-regulated or disrupted after the activation of Wnt signaling. The most effective way this occurs is through the binding of Fz to LRP5/6, which will disrupt the destruction complex before it can begin [59]. Our analysis showed an increased methylation of LRP5 within parous women, which suggests a decreased expression of LRP5/6 and a decreased cellular capability to stop the beta-catenin destruction complex in this way. The beta-catenin destruction complex begins with the binding of GSK3 to Axin, which leaves GSK3’s active site open to phosphorylate beta-catenin. Once phosphorylated, beta-catenin is ubiquitinated and sent to the proteasome for removal [59]. It is suggested that initial tumor development requires rapid and effective repression of GSK3B [58]. In our analysis through IGV, GSK3B was found to have a DMR hypermethylated in the nulliparous samples. This suggests an increase in expression of GSK3 within parous women and subsequently an increase in the activity of the beta-catenin destruction complex. 

PPP2CA, found to be hypermethylated within parous women, is also closely involved in canonical Wnt signaling. While the effect of PPP2CA in this context is still unclear, research leans toward a positive ability to stabilize beta-catenin [59]. The parous hypermethylation of PPP2CA, which suggests a lower expression in parous women, supports the idea of decreased beta-catenin. 

The noncanonical Wnt/calcium pathway, which is also found to be up-regulated in parous women as a result of increased FZD1 expression, occurs independently of beta-catenin. However, the noncanonical Wnt/calcium pathway is an inhibitor of canonical Wnt/beta-catenin signaling further along the line by stopping the transcriptional efforts of beta-catenin in the nucleus [60]. This inhibition occurs in one of two ways. The first uses the CaMKII-TAK1-NLK pathway, which inhibits beta-catenin-TCF-dependent transcription through the phosphorylation of TCF. The second uses NFAT-mediated transcriptional regulation to suppress beta-catenin-dependent-transcription.

Whereas more mechanistic studies need to be done in human breast cells, the data analyzed thus far indicate that the methylation of genes involved in Wnt signaling pathway could be another path involved in the protective effect of pregnancy in the human breast. 

## 6. Conclusions

Our work [22,23,27] clearly demonstrates that the breast of parous postmenopausal women exhibits a specific signature that has been induced by a full term pregnancy. This signature reveals for the first time that the differentiation process is centered in chromatin remodeling and the epigenetic changes induced by methylation of specific genes, that are important regulatory pathways induced by pregnancy. Through the analysis of the genes found to be differentially methylated between women of varying parity, multiple positions at which beta-catenin production and use is inhibited were recognized. First, the ability of the Fz receptor to bind to LRP5/6 and disrupt the beta-catenin destruction complex was down-regulated by a decrease in LRP5. Then, an increase in GSK3B suggests a strong up-regulation of the beta-catenin destruction complex, wherein GSK3B is responsible for marking beta-catenin for deletion. Third, a decrease in PPP2CA lowers its ability to stabilize beta-catenin. All of these transpire to decrease the amount of beta-catenin able to make it through the cytosol and into the nucleus. Once in the nucleus, however, the increased expression of the noncanonical Wnt/calcium signaling pathway interferes with the ability to beta-catenin to bind to TCF and help in transcription and EMT. The added effect of all of these differential methylations leans toward the conclusion that beta-catenin, especially as it pertains to the Wnt signaling pathway, is regulated differently between parous and nulliparous women. The decrease in beta-catenin production and accumulation may be a leftover effect from mammary involution, which would have been the last process of remodeling the mammary glands had undergone. This suggests that the decreased capacity for beta-catenin accumulation caused by involution is what causes the protective effect of pregnancy against breast cancer. The biological importance of the pathways identified in this specific population cannot be sufficiently emphasized due to the fact that they could represent another safeguard mechanism besides the ones discussed earlier [27], mediating the protection of the breast conferred by full term pregnancy.

## Supplementary Files

Supplementary File 1 Supplementary Materials (PDF, 910 KB)
