# Supplementary material for: The Genomic Signature of Breast Cancer Prevention
Source: Genes (Basel). 2014 Feb 26;5(1):65–83. doi: 10.3390/genes5010065 (PMC3978512; doi:10.3390/genes5010065)
Supplement: Supplementary File 1 — Supplementary Materials (PDF, 910 KB) [file genes-05-00065-s001.pdf]

Supplementary Materials

Figure S1. DMRs for the GSK3B and IL6ST genes. Gene shape can be seen above. The exons are shown as thicker blue bars within the gene shape. Areas marked as genetic introns are shown as a blue line with arrows to indicate the direction of the read. GSK3B's DMR (differentially methylated region) is opposite to the statistical analysis, which expects that the gene is parous hypermethylated overall. IL6ST is methylated within the promoter region, which may cause it to more greatly affect the overall gene regulation and function of the gene. Under the gene name is the direction of methylation followed by the exact location of the DMR within the chromosomal base pairs.

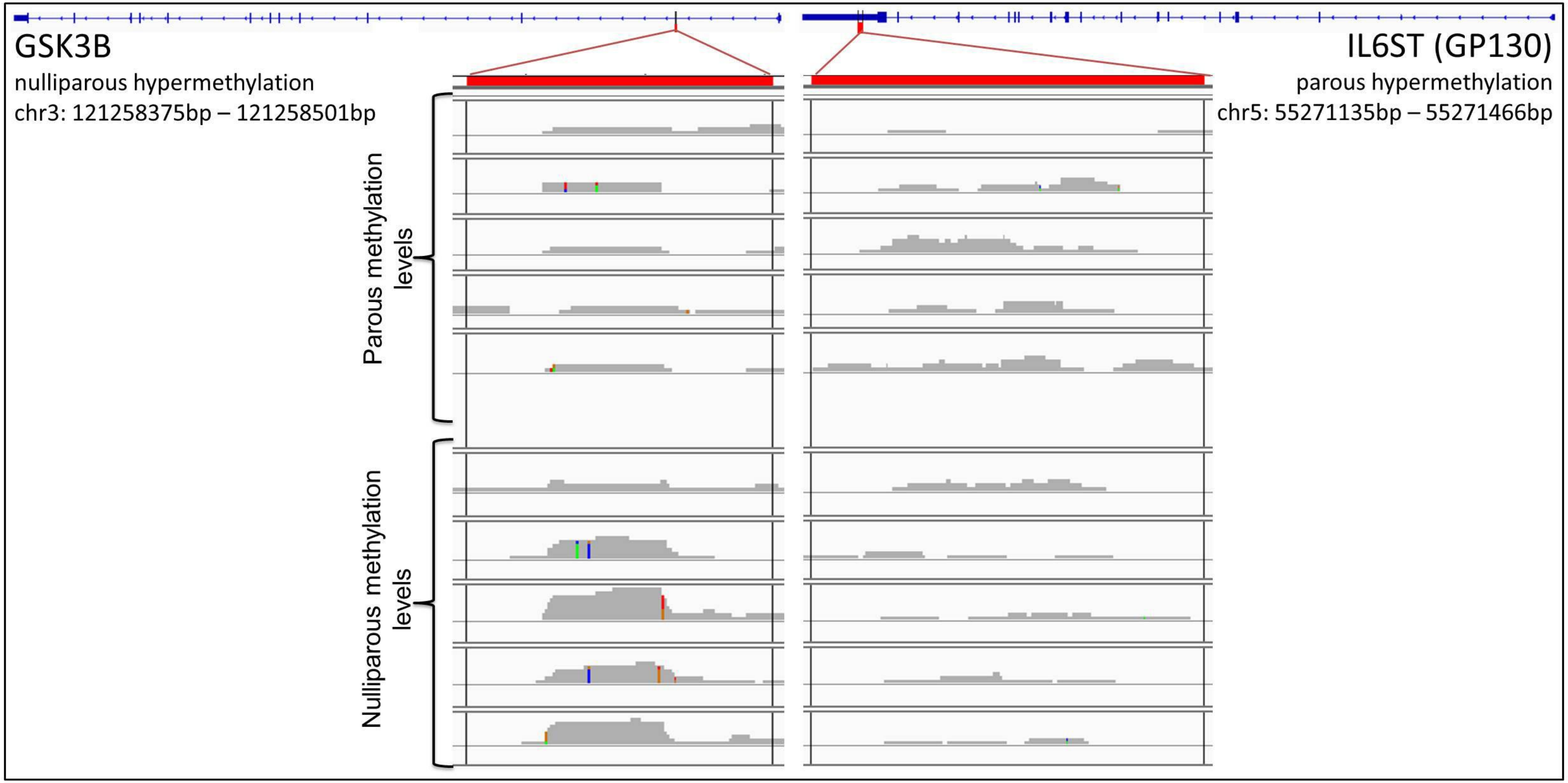

Figure S2. DMRs for DACT1 and ROBO1. Both genes are hypermethylated in the parous samples, which suggests a down-regulation of the gene within parous women.

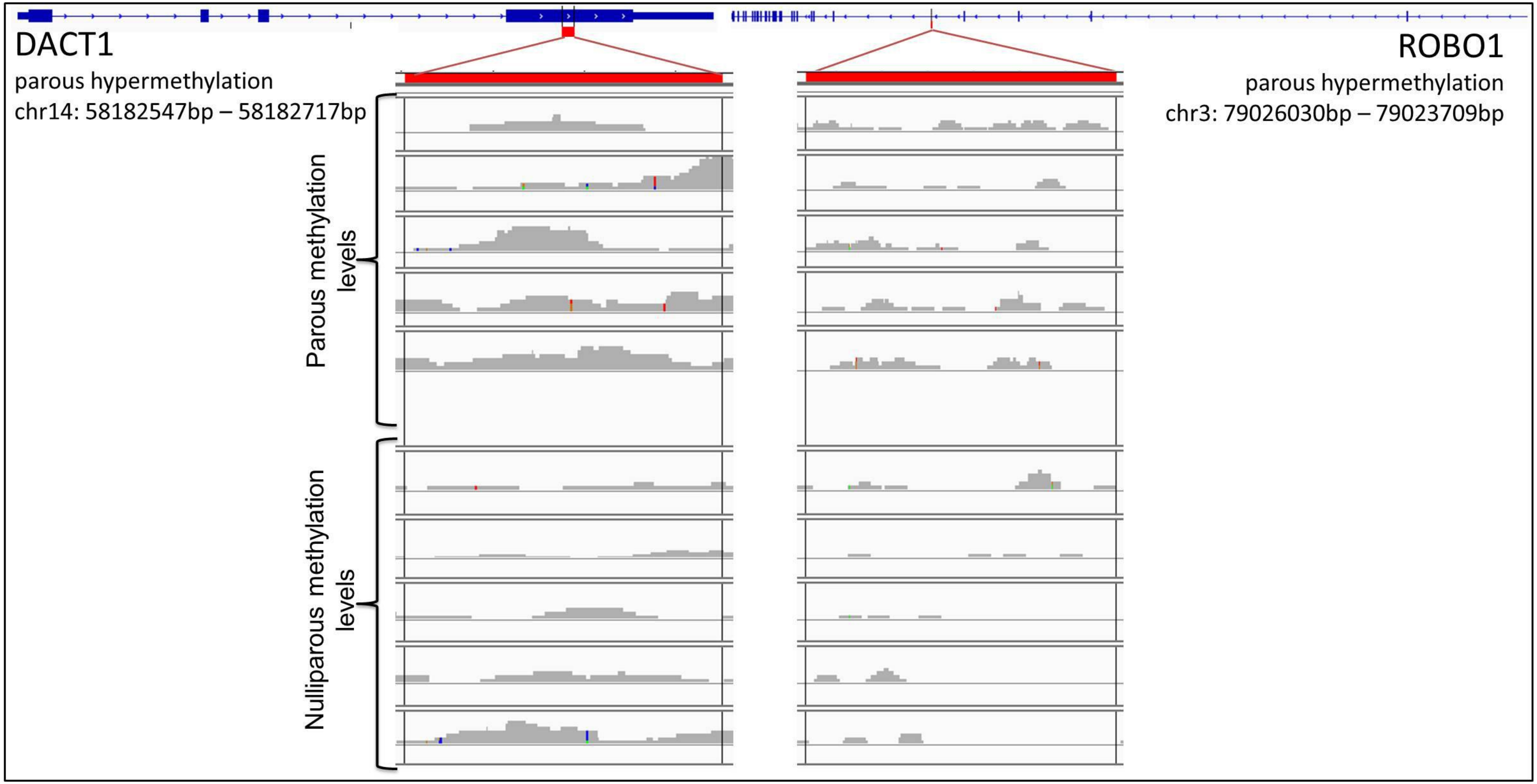

Figure S3. DMRs for INPP4B. The gene has three separate areas of differential methylation, all three have higher methylation levels in parous samples.

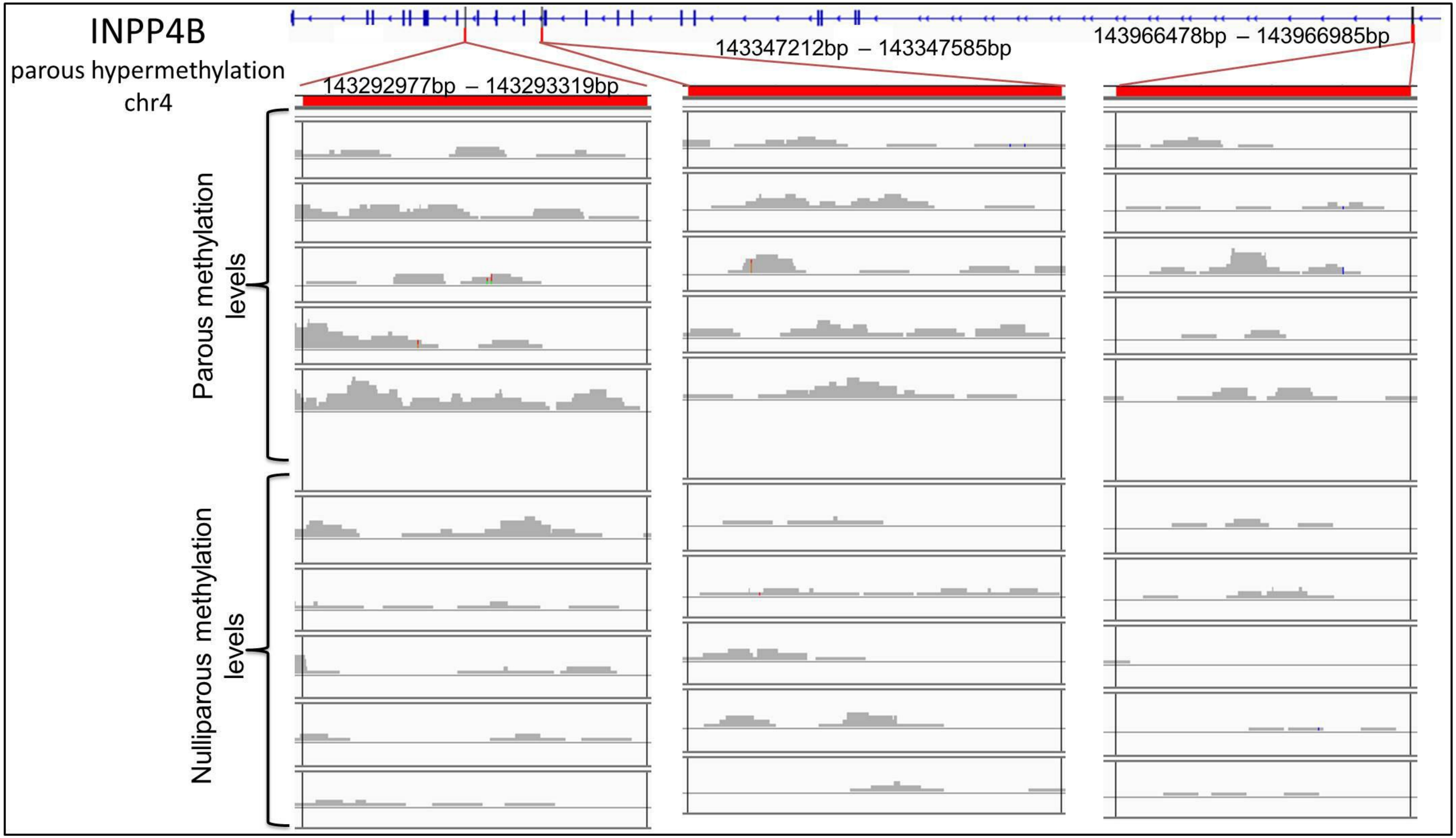

Figure S4. DMRS for FZD1 and PPP2CA. PPP2CA is hypermethylated in parous breast. FZD1 is hypermethylated in nulliparous, which suggests an up-regulation of the gene within women who have had a full term pregnancy.

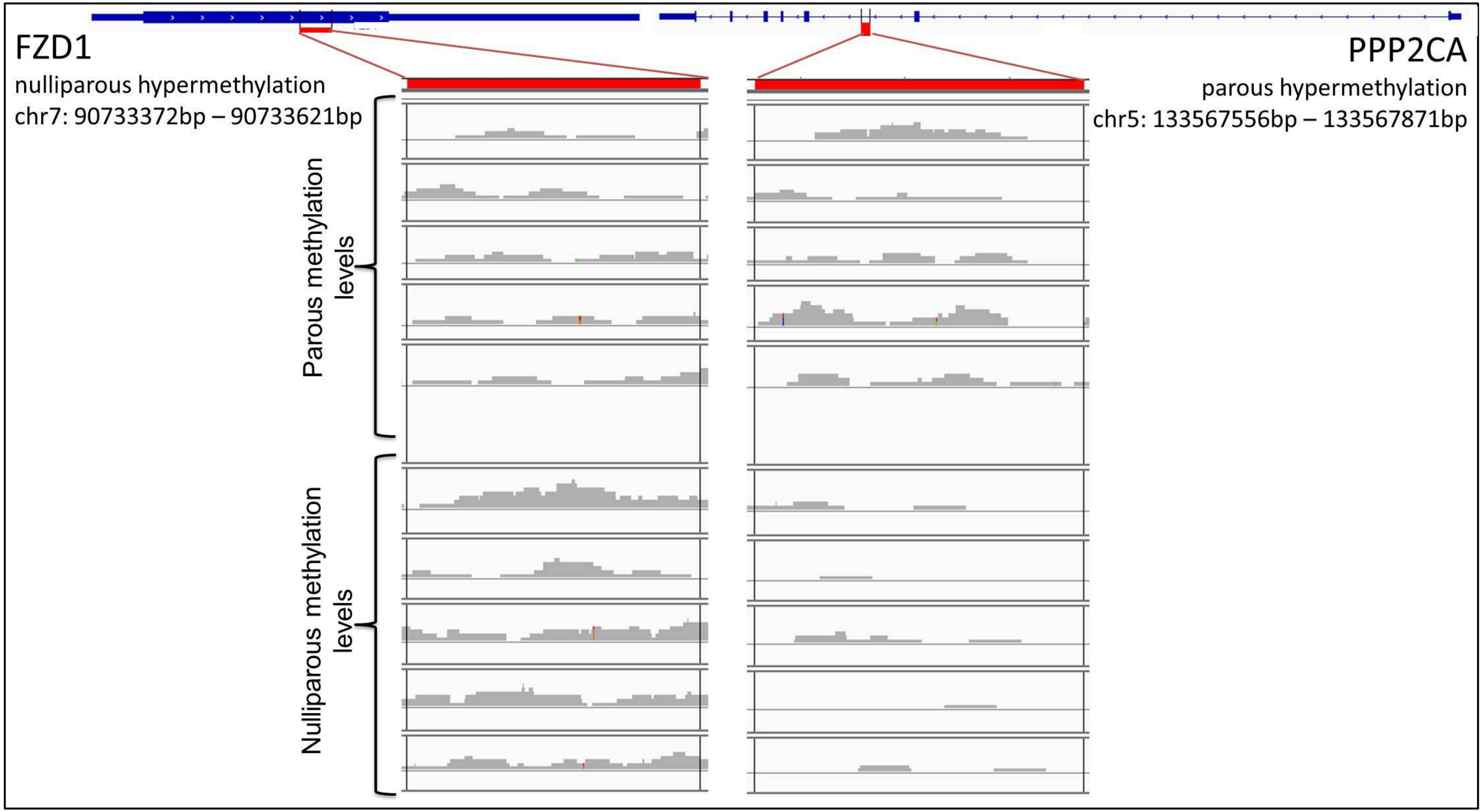

© 2014 by the authors; licensee MDPI, Basel, Switzerland. This article is an open access article distributed under the terms and conditions of the Creative Commons Attribution license (http://creativecommons.org/licenses/by/3.0/).
